# Current and Emerging Markers and Tools Used in the Diagnosis and Management of Chronic Kidney Disease–Mineral and Bone Disorder in Non-Dialysis Adult Patients

**DOI:** 10.3390/jcm12196306

**Published:** 2023-09-30

**Authors:** Maria Fusaro, Luciano Pereira, Jordi Bover

**Affiliations:** 1 National Research Council (CNR)—Institute of Clinical Physiology (IFC), Via G. Moruzzi 1, 56124 Pisa, Italy; 2 Department of Medicine, University of Padova, Via Giustiniani, 2, 35128 Padova, Italy; 3 Institute of Investigation and Innovation in Health, University of Porto, 4200-135 Porto, Portugal; 4 INEB—National Institute of Biomedical Engineering, University of Porto, 4150-180 Porto, Portugal; 5 DaVita Kidney Care, 4200-448 Porto, Portugal; 6 Faculty of Medicine, University of Porto, 4200-250 Porto, Portugal; 7 Nephrology Department, University Hospital Germans Trias i Pujol (HGiTP), 08916 Barcelona, Spain

**Keywords:** chronic kidney disease–mineral and bone disorder, secondary hyperparathyroidism, biomarker, calcification, bone, calcium, phosphate, PTH, vitamin D, osteoporosis

## Abstract

Chronic kidney disease (CKD) is a significant public health concern associated with significant morbidity and has become one of the foremost global causes of death in recent years. A frequent comorbidity of CKD is secondary hyperparathyroidism (SHPT), exemplified by high serum parathyroid hormone (PTH) levels. The mineral metabolism disturbances resulting from CKD and progression to SHPT are currently considered part of the definition of chronic kidney disease–mineral and bone disorder (CKD-MBD). However, CKD-MBD does not only include abnormalities in laboratory-measured parameters; it is a complex condition characterized by dysregulation of bone turnover, mineralization, growth and strength, accompanied by vascular or another soft-tissue calcification. Together, this increases the risk of bone fractures, cardiovascular disease, and overall mortality in CKD-MBD patients. Monitoring serum markers is essential in diagnosing SHPT and CKD-MBD, and there are several recognized indicators for prognosis, optimal clinical management and treatment response in late-stage kidney disease patients receiving dialysis. However, far fewer markers have been established for patients with non-dialysis CKD. This review provides an overview of current and emerging markers and tools used in the diagnosis and management of CKD-MBD in non-dialysis adult patients.

## 1. Introduction

### 1.1. CKD-MBD/SHPT

Chronic kidney disease (CKD) is a significant public health concern associated with significant morbidity. It has become one of the foremost global causes of mortality over the past 30 years [1]. An estimate from 2017 suggests that CKD affects up to nine percent of the global population, and CKD is predicted to become the fifth leading cause of premature mortality worldwide by 2040 [1,2]. Complex disturbances to mineral metabolism and dysregulation of homeostasis are associated with declining kidney function in CKD [3,4,5], and a frequent complication is secondary hyperparathyroidism (SHPT), defined by elevated serum parathyroid hormone (PTH) and enlargement of the parathyroid glands [6].

SHPT is an adaptive and typically detrimental condition that develops due to declining kidney function and the subsequent dysregulation of calcium and phosphate homeostasis. This leads to increased serum phosphate, elevated fibroblast growth factor-23 (FGF-23) levels, and reduced synthesis of klotho (the FGF-23 co-receptor) and the active form of vitamin D, calcitriol. These alterations directly and indirectly cause elevated production and secretion of PTH and parathyroid hyperplasia, further exacerbating the condition [3]. The presence of multifactorial hyporesponsiveness to PTH creates an additional negative feedback loop [7].

The mineral metabolism disturbances resulting from CKD and rising PTH levels are currently considered part of the definition of chronic kidney disease–mineral and bone disorder (CKD-MBD) [4]. However, CKD-MBD does not only include abnormalities in laboratory-measured parameters; it is a complex condition characterized by dysregulation of bone turnover, growth, mineralization, or strength, accompanied by soft tissue and vascular calcification (VC) [4,8,9]. Together, this increases the risk of bone fractures, cardiovascular disease, and overall mortality in CKD-MBD patients. Consequently, bone is now considered a novel endocrine organ at the center of CKD-MBD [10]. Moreover, the involvement of bone metabolism in CKD-MBD has clear parallels for cardiovascular health and aging in the general population [11].

Monitoring of serum markers, including calcium, phosphate, PTH, vitamin D and alkaline phosphatase activity, as well as other diagnostic tools, are essential in the management of SHPT and CKD-MBD, and treatment decisions should be based on a combined assessment of these parameters [4]. Although FGF-23 could represent an early indicator of phosphate overloading, and klotho may be an important preliminary marker of CKD, monitoring these proteins is not yet recommended in regular practice [4].

### 1.2. Diagnostic Tools in CKD-MBD

Biochemical anomalies are common in CKD [12,13], and, as previously highlighted, the diagnosis of CKD-MBD typically includes laboratory testing for serum PTH, calcium, phosphate and alkaline phosphatase activity [4,9]. Fluctuations in the biochemical profile of CKD-MBD may be evident in CKD stage 3 or earlier for parameters not typically measured in regular practice [4,13], such as decreased levels of klotho [14]. However, the rate of change and severity of anomalies may vary among patients [12]. Given the complexity of variation within any one parameter, trends of changes are more important than individual values to assess the severity of abnormal laboratory results in CKD-MBD and inform therapeutic decisions [4].

The benchmark for diagnosis of renal osteodystrophy is bone biopsy with histomorphometric analysis, which is the only technique capable of providing a full evaluation of dynamic bone parameters [15,16]. However, the use of bone biopsy has decreased in recent years due to a lack of proficiency in performing the procedure and interpreting the findings, perceived invasiveness of the technique and corresponding pain for patients, and issues surrounding healthcare reimbursement [15,16]. Recent updates to clinical guidelines recommend assessment of bone mineral density (BMD) by dual-energy X-ray absorptiometry (DXA) at the spine or hip for diagnosis of osteoporosis [4,17]; however, imaging should not be considered a complete surrogate for histological findings as it is not sensitive enough to fully evaluate underlying mineral and bone disorders [15,16].

For the diagnosis of cardiovascular calcifications in CKD-MBD, lateral abdominal radiography is recommended to detect VC, while an echocardiogram can be used to assess the degree of valvular calcification [4,18]. Other techniques, such as simple radiography of the hands and pelvis and pulse wave velocity, can provide additional helpful information [8,19]. More detailed information on the diagnosis of CKD-MBD is beyond the scope of this paper and is reviewed in other articles [11,20,21,22,23].

### 1.3. Objective

**Figure 1:**
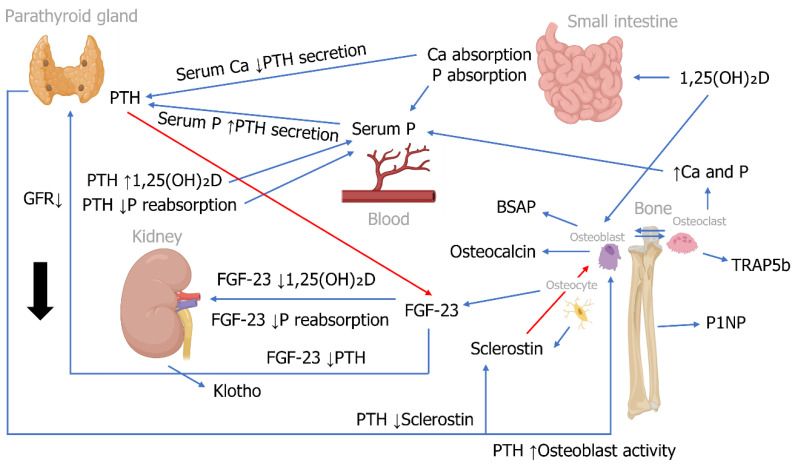
Schematic of key CKD-MBD marker interactions (adapted from Miller PD. Bone Res 2014 and created using images from BioRender.com).

**Figure 2:**
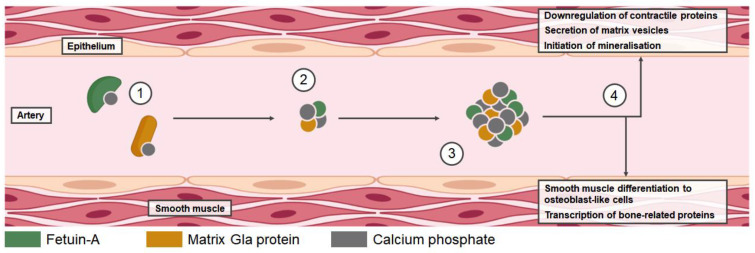
Mechanism of medial calcification. (1) Under baseline physiological conditions, fetuin-A and Matrix Gla proteins can remove calcium–phosphate complexes; (2) In CKD, elevated calcium phosphate levels can saturate regulatory proteins, and the interaction between fetuin-A and Gla proteins initiates the formation of amorphous primary calciprotein particles (CPPs); (3) Aggregation of primary CPPs leads to the formation of crystalline secondary CPPs; (4) Secondary CPPs contribute to conditions that induce differentiation of smooth muscle cells into osteoblast-like cells, which then express osteoblast transcription factors and other bone-related proteins. Contractile proteins are downregulated, accompanied by the secretion of mineralization-competent extracellular matrix vesicles that induce the calcification within blood vessel walls.

There are several recognized markers for prognosis, optimal clinical management and treatment response in late-stage kidney disease patients receiving dialysis; however, fewer markers have been established for CKD patients not receiving dialysis. This review aims to provide an overview of current and emerging markers or risk factors for disease progression, cardiovascular dysfunction, and bone turnover in non-dialysis patients with CKD-MBD. Markers described include parameters that can be assessed via blood samples. The functions of markers typically detected in systemic circulation are summarized in Table 1, and a schematic summarizing some key marker interactions can be found in Figure 1. Figure 2 describes interactions between cardiovascular dysfunction markers. In addition, this review highlights some of the diagnostic tools available for the assessment of cardiovascular and bone health (Table 2).

## 2. General Markers of Mineral Dysregulation

Abnormalities of mineral metabolism are very common in patients with CKD. As previously mentioned, variations in laboratory-measured levels of circulating markers may begin in CKD stage 3 or earlier, and monitoring is essential for effective management [4]. The following section summarizes general markers associated with adverse patient outcomes in non-dialysis CKD.

### 2.1. Phosphate

Although monitoring of serum phosphate levels is standard practice for the diagnosis and management of CKD-MBD, there is evidence to suggest a role for phosphate as a marker of disease progression and outcomes. For example, research has shown that high serum phosphate levels in non-dialysis CKD patients are associated with renal function decline and an increased risk of end-stage kidney disease and death [24,25,26,27]. The recent PECERA study identified the minimum risk ranges of serum phosphate for all-cause mortality (2.8–5.0 mg/dL) and cardiovascular mortality (2.1–4.4 mg/dL) [28], which correlate well with the ‘normal’ range indicated in current guidelines [4]. Values exceeding these ranges are associated with an increased risk of mortality [28]. Accordingly, current KDIGO (Kidney Disease Improving Global Outcomes) guidelines suggest reducing elevated phosphate levels ‘towards’ the normal range in CKD patients and basing decisions about phosphate-modulating treatment on progressively or persistently elevated serum phosphate [4].

### 2.2. Parathyroid Hormone

Parathyroid hormone is a peptide secreted by the parathyroid glands that regulates serum calcium concentration through interaction with the kidneys, bones and gastrointestinal tract [24]. The active biological form is called ‘intact’ PTH (iPTH) and contains 84 amino acids secreted after cleavage from a larger inactive precursor [25,29]. Elevated iPTH is a hallmark of CKD and SHPT. In this setting, PTH is considered a uremic toxin which causes harmful complications, including bone loss, soft tissue calcification and cardiomyopathy, among many others [25]. Substantially elevated PTH is typically observed in patients requiring imminent dialysis therapy [25]; however, PTH levels may rise as early as in stage 3 CKD [13,27]. Correspondingly, several studies have observed that elevated PTH correlates with declining renal function in patients with non-dialysis, stage 3–5 CKD [26,28,30,31]. Additionally, results from observational studies have shown that PTH is an independent predictor of fractures, vascular events and death in adult patients with CKD stage 3 and 4 CKD [32] and that rising PTH levels may be linked to the progression of CKD and an increased incidence of cardiovascular events [33,34]. Nevertheless, despite evidence linking elevated iPTH levels with adverse outcomes, there is currently limited data to indicate that reducing iPTH in non-dialysis patients leads to a reduced risk of adverse outcomes.

While a consensus exists that rising PTH levels serve as an indication for treatment initiation, it has been suggested that complete normalization of PTH levels is to be avoided [35,36,37], due to the beneficial phosphaturic effects of PTH, the presence of hypo-responsiveness to PTH in CKD and to avoid low turnover bone disease. For instance, in dialysis patients, iPTH levels between 2–9 times the upper limit of normal, or increasing trends between those values, is the suggested level at which iPTH should be maintained [4,8]. In contrast, the latest KDIGO guidelines could not define the optimal PTH level in non-dialysis CKD patients [4] due to a lack of robust evidence. This issue has been recently reviewed in other articles [35,37].

There is a balance to be found when managing rising PTH levels, as parathyroid hyperplasia, persistent elevation of PTH, and the progression of SHPT resulting from delayed therapeutic interventions are accompanied by continuing reductions in sensitivity to vitamin D and calcium homeostasis, subsequently posing a risk of treatment resistance in later stages of the disease [6]. Additionally, patients starting hemodialysis with iPTH > 600 pg/mL have a high risk of maintaining substantially elevated iPTH levels after one year of receiving hemodialysis compared with patients starting dialysis with lower iPTH levels. This may occur despite the more aggressive treatments prescribed to the patients starting hemodialysis with iPTH > 600 pg/mL [38]. This further suggests the need for effective PTH control to avoid progressive SHPT during non-dialysis CKD and raises the need for a consensus on PTH target guidelines in these patients [38].

Despite some of the challenges with PTH measurement, it remains a key marker for monitoring CKD-MBD [39]. As mentioned later in this review, PTH combined with other markers has been assessed, but mainly in dialysis patients [40], and requires further research.

### 2.3. Vitamin D

Individuals can obtain vitamin D through multiple sources: endogenous synthesis from UV exposure to the skin, dietary intake, or supplementation. Upon entering the bloodstream, vitamin D undergoes hepatic hydroxylation, resulting in the formation of 25(OH)D (calcidiol or calcifediol). 25(OH)D is biologically less active compared to 1,25(OH)_2_D (calcitriol), which is generated in the kidney after another hydroxylation step. In clinical settings and epidemiological studies, 25(OH)D is the most widely used marker of vitamin D status [41]. Calcitriol is not frequently used as a marker as the assays developed to measure 1,25(OH)_2_D lack standardization, its short half-life complicates accurate measurement, and exogenous administration of calcitriol and vitamin D analogs can artificially influence results [8].

Vitamin D deficiency (defined by most experts as serum 25(OH)D serum levels <20 ng/mL) [42] is common in CKD and can affect more than 80% of non-dialysis patients [43]. Several studies provide evidence suggesting a robust inverse association between serum 25(OH)D levels and poor outcomes in CKD patients not receiving dialysis. For example, low levels of 25(OH)D have been associated with deteriorating renal function and an elevated risk of mortality in non-dialysis CKD [29,44,45,46].

Guidelines differ regarding recommendations to supplement low 25(OH)D levels. Australian and US guidance states that levels < 30 ng/mL should be corrected using appropriate treatment strategies even in earlier stages of CKD, whereas KDIGO advised that adult non-dialysis patients with stage 3–5 CKD and vitamin D deficiency should be treated with nutritional vitamin D. In contrast, calcitriol and vitamin D analogs should not be routinely prescribed, reserving these for stage 4–5 CKD patients with severe and progressive hyperparathyroidism [4,47]. However, a consensus from the National Kidney Foundation defined ‘adequate’ 25(OH)D concentrations in CKD stages 3–4 as >20 ng/mL, assuming the absence of a counter-elevation in PTH levels, and agreed that 25(OH)D concentrations < 15 ng/mL should be corrected regardless of PTH level [32].

### 2.4. Calcium

Several studies have identified relationships between dysregulation of serum calcium levels and outcomes in non-dialysis patients. For example, results from two observational studies show that low serum calcium levels are associated with renal function decline and progression to renal replacement therapy. Together, these studies suggest that serum calcium could be considered in a predictive model of patient prognosis [48,49]. A more recent study of patients with stages 4–5 non-dialysis CKD showed an increased risk for all-cause mortality at serum calcium levels > 10.5 mg/dL. The lowest risk range for cardiovascular mortality was between 8.5–10.0 mg/dL, and concentrations on either side of this threshold were likely to correspond to a higher cardiovascular mortality [33]. Current guidelines recommend that hypercalcemia is to be avoided in adult patients with stage 3–5 CKD but suggest an individualized strategy with regard to treatment rather than endorsing the correction of hypocalcemia for all patients [4], as serum calcium levels are not necessarily a reflection of total calcium burden and high phosphate levels are usually treated first.

### 2.5. FGF-23

FGF-23 is a 32 kDa glycoprotein mainly produced in bone by osteoblasts and osteocytes. The primary action of FGF-23 is the inhibition of phosphate reabsorption from the urine and the suppression of calcitriol synthesis in the kidney [50]. FGF-23 may represent an important marker in non-dialysis CKD, as several studies have linked serum FGF-23 levels to adverse patient outcomes.

Increases in serum FGF23 have been demonstrated as one of the early indicators of mineral dysregulation in non-dialysis patients, compared with phosphate and PTH, with high FGF-23 and low 25(OH)D levels identified as independent predictors of poor renal outcome [34]. Furthermore, a prospective study of 3879 participants with stages 2–4 CKD found that elevated FGF-23 was an independent risk factor for end-stage kidney disease (ESKD) and progression to dialysis in patients with relatively preserved kidney function, and for mortality across all stages of CKD enrolled [51].

In a later study, elevated FGF-23 was associated with risk of progression to renal replacement therapy (hazard ratio [HR] 1.35; 95% confidence interval [CI] 1.001–1.820; *p* = 0.05), as well as both fatal and non-fatal cardiovascular events (HR 1.74; 95% CI 1.303–1.305; *p* < 0.001) and all-cause mortality (HR 1.4; 95% CI 1.109–1.767; *p* = 0.005) [52]. Similar outcomes were observed in a large meta-analysis of 15 prospective cohort studies, where elevated FGF-23 levels in non-dialysis patients with CKD stages 1–5 were associated with an increased risk of all-cause mortality (risk ratio [RR] 1.46; 95% CI 1.38–1.55; *p* < 0.001), cardiovascular disease (RR 1.37; 95% CI 1.15–1.63; *p* < 0.001), and renal events (RR 1.31; 95% CI 1.07–1.59; *p* = 0.008), including progression to ESKD or the initiation of dialysis [53]. Finally, two smaller investigations highlighted that raised serum FGF-23 correlates with levels of bone metabolism-related markers and vertebral fractures in older non-dialysis patients, as well as increased insulin resistance in non-diabetic, non-dialysis stage 3–5 CKD patients [44,54], among many other potential pleiotropic effects [45].

### 2.6. Klotho

Klotho is a protein produced mainly in the kidney that can exist in a membrane-bound state or a soluble, circulating form [46]. The membrane form of klotho acts as a co-receptor for FGF-23. Soluble klotho is found in the blood, urine and cerebrospinal fluid and carries out a range of functions, including the suppression of growth factor signaling and the regulation of ion channels and transporters [55,56]. Klotho gained attention due to its association with extending survival [46,57,58] and subsequently as an early biomarker of acute or chronic kidney injury [59]; however, initial studies in CKD provided contradictory results regarding deterioration of renal function and increased mortality [60,61].

Evidence shows that a decline in serum klotho level can occur as early as CKD stage 2 and continues to reduce as CKD progresses [62,63,64]. Recent studies have also suggested that in patients with non-dialysis CKD, lower soluble Klotho levels in combination with higher FGF-23 levels are associated with adverse clinical outcomes, including overall mortality and cardiovascular events in non-diabetic non-dialysis CKD patients [55,65]. In addition, analyses have shown an association between decreased serum klotho levels and the risk of vascular calcification [66,67]. These results hint that klotho could be a promising CKD diagnosis and prognosis marker. However, evidence relating to the clinical significance of klotho in CKD patients is inconsistent, and therefore, this topic requires further study [68].

## 3. Diagnostic Tools and Markers of Cardiovascular Dysfunction

### 3.1. Vascular Calcification in CKD-MBD

Vascular calcification (VC) is a highly complex process involving multiple signaling pathways; there are many promoters as well as inhibitors of calcification [69]. There are two main types of VC: intimal calcification and medial calcification (Mönckeberg’s sclerosis) [70,71]. Intimal calcification is linked to atherosclerosis, a condition characterized by chronic inflammation and/or lipid accumulations [71,72]. Medial calcification is a form of arteriosclerosis and occurs in the elastic region of the arteries without involvement of lipid or inflammatory components [71,73]. Calcium and phosphate ions play a key role in the initiation of medial calcification in CKD, forming circulating calcium–phosphate nanoparticles (Figure 2). Regulatory proteins can remove calcium–phosphate complexes under normal conditions but can eventually become saturated in CKD, leading initially to the formation of primary calciprotein particles (CPPs) followed by conversion to secondary CPPs [74]. CPPs contribute to conditions that induce the differentiation of vascular smooth muscle cells into osteoblast-like cells, which subsequently express osteoblast transcription factors and other bone-related proteins. This corresponds with the downregulation of contractile proteins and secretion of matrix vesicles, which trigger mineralization within blood vessel walls [69]. Medial and intimal calcification occur in CKD, but medial calcification is more common [70,75].

VC is associated with several adverse clinical outcomes, including cardiac events, cardiovascular mortality and all-cause mortality [76]. Guidelines indicate that patients with stage 3–5 CKD and evident vascular or valvular calcification have the greatest cardiovascular risk, and it is sensible to use this knowledge to inform the management of CKD-MBD [4], for instance, via reducing the use of hypercalcemic/hyperphosphatemic drugs or restricting calcium-based phosphate binders.

Patients with CKD exhibit an elevated cardiovascular risk, and cardiovascular disease, rather than ESKD, is the leading cause of death in patients with advanced CKD. In CKD patients not receiving dialysis, the incidence and prevalence of cardiovascular events are also significantly higher compared with the general population [77]. This section highlights the current evidence surrounding markers of cardiovascular events and cardiovascular dysregulation tools in non-dialysis CKD populations.

### 3.2. Vascular Calcification Imaging Tools

Several imaging methods have been developed to evaluate the extent and severity of VC in patients with CKD, many of which do not differentiate between intimal and medial calcification [78].

Coronary artery calcification (CAC) scoring is a technique to assess the likelihood of coronary heart disease and mortality in the general population [49], and is typically quantified by computed tomography (CT) [79,80]. Studies have indicated that CAC may be present even in mild-to-moderate non-dialysis adult CKD patients [81], and is linked to an increased risk of declining kidney function [82]. Additionally, high CAC scores have been linked with faster progression to ESKD and mortality [83,84], with a meta-analysis of eight studies involving 862 subjects identifying a considerable association between high CAC and cardiovascular events in non-dialysis patients [85].

While the CAC score is the gold standard to measure VC, the technique delivers high radiation doses and can incur significant costs [86]. Consequently, guidelines suggest that simpler radiographical methods, such as lateral abdominal radiography, can be used as reasonable alternatives to CT-based methods to identify the degree of VC [4].

The Kauppila score is a validated grading system used to assess the severity of abdominal aortic calcification based on lateral abdominal radiography [18]. Aortic VC scores using this system correlate significantly with renal resistive index (RRI) in non-dialysis CKD patients, suggesting it could be a convenient and inexpensive tool for estimating RRI and, consequently, the intrarenal vascular status [87]. However, further research is warranted [87]. Furthermore, the degree of calcification in the abdominal aorta, measured by the Kauppila score, has been strongly associated with a decline in kidney function and an increase in adverse cardiovascular parameters, including pulse pressure, left ventricular mass, left atrial volume, and left atrial volume index in CKD patients not receiving renal replacement therapy [88].

Adragao scoring is another simple VC score based on plain radiographical imaging of the pelvis and hands [19]. VC assessment using the Adragao score has also been shown to independently predict death and time to hospitalization in non-dialysis CKD patients. Consequently, it may represent a useful index to detect patients with CKD at high risk of complications or death [89]. Recently, a small study utilizing a plain X-ray of the lumbar spine, pelvis, and hands to assess VC reported that bone formation rate was significantly lower in patients with VC than patients without VC. This was the first study reporting a relationship between the histomorphometric qualities of bone and simple VC scores in non-dialysis patients [90].

Kauppila and Adragrao scoring are semi-quantitative, relying on a visual assessment and, therefore, are more susceptible to human errors. Recently, an X-ray-based, computer-assisted abdominal aorta VC score has been developed with improved precision versus the Kauppila score. Ongoing studies will determine whether this new scoring method may be beneficial in detecting the progression of VC [86]. Finally, breast arterial calcification (BAC) is becoming well-recognized as a specific marker of medial calcification and is commonly observed in CKD patients, increasing in prevalence and severity with disease progression [91]. Therefore, measurement of BAC may offer a personalized, non-invasive approach to differentiate women at risk for cardiovascular disease, with no additional exposure to radiation or financial implications, as most women aged 40 years or above experience routine breast cancer screening via mammography [91].

### 3.3. Pulse Wave Velocity

Pulse wave velocity (PWV) is a non-invasive method for analyzing central arterial stiffness based on the rate at which blood pressure pulses travel through the circulatory system. It is a straightforward and reproducible technique and is validated and widely used in clinical settings throughout Europe. Changes in PWV have independent predictive value for cardiovascular risk in the general population [92].

In non-dialysis patients with CKD, several studies have correlated high PWV, indicative of increased arterial stiffness, with deterioration of renal function [93,94,95]. Additionally, data from an extensive cross-sectional study in 1385 non-dialysis patients with CKD (KNOW-CKD cohort) indicated that greater arterial stiffness, indicated by elevated PWV, corresponded with coronary artery calcification [96]. Correspondingly, two recent clinical studies identified arterial stiffness as an important predictor of cardiovascular events and risk [97,98], whereas a meta-analysis of studies in non-dialysis patients showed that high PWV was also associated with an increased risk (RR 2.52; 95% CI 1.40–4.55; I^2^: 62.6%) of all-cause mortality [85]. Together, these observations suggest that monitoring arterial stiffness via PWV may represent an inexpensive method for estimating the extent of disease progression and likelihood of death in non-dialysis patients with CKD.

### 3.4. Regulators of Vascular Calcification

Fetuin-A is a multifunctional, circulating glycoprotein that scavenges calcium phosphate via negatively charged amino acids β-sheet of the N-terminal domain [99,100,101]. Matrix Gla protein (MGP) and Gla-rich protein (GRP) also scavenge calcium phosphate via their negatively charged γ-carboxylated glutamate residues. The interaction between fetuin-A and Gla proteins initiates the formation of primary CPPs [101], which help regulate VC [101]. Fetuin-A, MGP and CPPs offer potential as markers of cardiovascular health in non-dialysis adult patients with CKD.

### 3.5. Fetuin-A

Low serum fetuin-A is typically linked to mineralization imbalance and increased likelihood of death in advanced kidney disease [102]. However, the situation in non-dialysis CKD is still unclear. Some studies have identified associations between low fetuin-A levels, accelerated progression of aortic VC and major adverse clinical events [103,104], whereas increased phosphorylated fetuin-A-containing CPPs have also been associated with increased aortic stiffness in patients with non-dialysis CKD [105]. Nevertheless, in a recent meta-analysis of 5169 CKD patients, a significant association between low fetuin-A levels and higher risk of mortality was observed in dialysis patients but not in the non-dialysis population [106].

### 3.6. MGP

Matrix Gla protein (MGP) is a powerful inhibitor of VC, and carboxylation by vitamin K is required to trigger the active, inhibitory state [107]. Correspondingly, markedly increased levels of inactive MGP have been observed in late-stage CKD patients and correlate with surrogate markers of VC and mortality [108]. However, the role MGP plays as a marker in non-dialysis patients is less well-known. For example, one study in patients with CKD stages 1–4 found no significant relationship between MGP and measures of arterial stiffness; however, this study did not differentiate between active and inactive forms of MGP [109]. Two other small studies in patients with CKD stages 2–5 not reliant on dialysis suggested that high levels of the inactive form of MGP are linked to kidney damage and may also serve as a marker of cardiovascular risk in CKD patients [110,111].

As an essential co-factor for MGP activation, poor vitamin K status may contribute to a high VC burden in CKD patients [112]. Vitamin K antagonist therapies, such as warfarin, can also contribute to vitamin K deficiency in CKD patients and further increase the risk of VC. Indeed, results from one cross-sectional study show that patients on hemodialysis treated with warfarin have an increased risk of VC [113]. Uncarboxylated (inactive) MGP reflects inadequate vitamin K-dependent carboxylation and may serve as a better predictor of cardiovascular risk in CKD patients than active MGP [114].

### 3.7. Primary and Secondary CPPs

Primary CPPs are small, spherical complexes of amorphous calcium phosphate and other regulatory proteins. Primary CPPs develop into larger, needle-shaped secondary CPPs containing crystalline calcium phosphate [115]. Our understanding of CPPs is evolving, but it is known that CPPs are associated with the development of VC in CKD patients, and clinical studies have shown that serum CPP levels increase as CKD progresses [116]. Methods for measuring serum CPP levels require standardization, and more research into their potential as a marker in CKD-MBD is warranted.

In addition, the T50 calcification inhibition test has recently been developed to determine the overall tendency for calcification of a patient’s serum. This in vitro diagnostic method evaluates the maturation of primary to secondary CPPs in serum, with T50 being the half-maximum time in minutes for this conversion [117]. As T50 provides information on the equilibrium point of calcification processes within the extracellular fluid, it could be considered a more compelling predictor of patient outcome than single protein or molecule measurements [118], such as fetuin-A or MGP. Serum calcification propensity has been independently associated with several cardiovascular outcomes, including myocardial infarction, peripheral vascular disease, heart failure, and all-cause mortality, in a large (*n* = 2785) study of patients receiving hemodialysis [119]. Additionally, a prospective cohort study in patients with stages 3 and 4 CKD reported that serum calcification propensity was independently associated with progressive aortic stiffening and an increased risk of all-cause mortality [118]. A further study of 3404 participants with stage 2–4 CKD found that a higher serum calcification propensity corresponded with atherosclerotic cardiovascular disease, ESKD, and mortality, independent of conventional risk factors for cardiovascular disease; however, this association was not independent of kidney function [120].

These studies suggest that serum T50 may be advantageous as a marker to monitor treatments against VC; however, future studies are needed to further evaluate T50 in non-dialysis patients and establish whether therapies to improve calcification promoter/inhibitor regulation might benefit patients [118,120].

### 3.8. Osteocalcin

Osteocalcin is a vitamin K-dependent hormone derived from osteoblasts and has roles in bone mineralization, bone cell activity, and regulation of glucose and lipid metabolism [121,122]. Although a marker of bone turnover, recent studies suggest that osteocalcin may also be associated with vascular health, including VC [122,123]. To date, few studies have assessed osteocalcin as a marker in non-dialysis CKD; however, some data have been reported.

Osteocalcin correlated negatively with renal function in a small study of non-dialysis CKD patients [124], and other investigations have identified a link to cardiovascular risks: a cross-sectional analysis of 256 non-dialysis CKD patients found that low levels of total osteocalcin were associated with endothelial dysfunction, whereas participants with high osteocalcin levels had a greater degree of arterial stiffness [125]. Additionally, a further study focusing on the uncarboxylated form of osteocalcin (ucOC) demonstrated that decreased ucOC levels were closely correlated with subclinical carotid atherosclerosis in non-dialysis CKD patients [126]. Further investigation is required, however, to ascertain if ucOC has utility as a novel marker for predicting atherosclerosis and cardiovascular disease in CKD patients not on dialysis.

## 4. Diagnostic Tools and Markers of Bone Dysregulation

### 4.1. Mechanism of Bone Deterioration

During normal kidney function, a reduction in calcium causes calcium-sensing receptors in the parathyroid glands to release PTH, causing the kidney’s reabsorption of calcium from urine into the blood and increased excretion of phosphate. PTH also stimulates the formation and release of calcitriol to increase calcium uptake from the small intestine to the blood, especially calcium release from bone, returning circulating calcium levels to the basal range and achieving homeostasis [3,8,127].

In CKD patients, when the glomerular filtration rate falls below normal retrievable levels, the kidneys cannot increase calcium uptake from urine to blood and stimulate the production of calcitriol to increase calcium absorption from the gut, leading to hypocalcemia. This further stimulates PTH secretion via calcium-sensing receptors, causing bones to release calcium (bone resorption) to regulate serum calcium levels, weakening them and causing symptoms including bone pain in the lower back, hips and legs, with a significant fracture risk correlating to increasing PTH levels [3,8,24,127].

These bone quality and strength abnormalities are observed in most patients with CKD stage 3–5 and in all patients treated with dialysis [128]. Consequently, patients with mild-to-moderate CKD are predisposed to a higher likelihood of fractures, and the risk of fracture rises as kidney function declines. Evidence suggests that many non-dialysis CKD patients experience adynamic bone disease with low bone turnover [129]. High-turnover bone disease develops later as CKD progresses to stages 4–5 D [129]. This section reviews current and emerging bone dysregulation tools and markers in non-dialysis CKD patients.

### 4.2. Alkaline Phosphatase

Serum alkaline phosphatase (ALP) is a hydrolytic enzyme that catalyzes the removal of phosphate groups from protein and nucleotides. Although it is produced by many tissues, it is typically concentrated in the bone and liver [130]. Bone-specific alkaline phosphatase (BSAP) is associated with the cell membrane of osteoblasts and is also partly released into the serum. BSAP stimulates tissue mineralization mainly through the inactivation of the mineralization inhibitors [131] and strongly correlates with bone formation rate [132]. High total ALP and BSAP levels are associated with increased mortality and fracture rate in hemodialysis patients [133,134,135].

In non-dialysis CKD patients, BSAP has been identified as an independent predictor of total hip BMD; furthermore, monitoring the B/I isoform of BSAP predicts osteopenia in the hip and the distal third of the radius in stage 3 CKD [136]. In another study, the authors suggested that in patients with non-dialysis stage 3–5 CKD, measurements of BMD were useful to demonstrate bone loss but not sensitive enough to distinguish the quantity of bone loss between different stages of CKD. Conversely, higher serum levels of BSAP and total ALP were observed in more advanced stages of renal failure and reflected an increased fracture risk of the femur [137]. A strong correlation has also been observed between serum ALP, iPTH and BMD [112,138], suggesting serum ALP may serve as a useful substitute for PTH as a biochemical marker to identify patterns of bone turnover [112]. An additional study suggested that the combined monitoring of BSAP and iPTH provides significant predicting power for non-invasive assessment of bone health in CKD and is a more reliable index than either marker used alone [139]. However, the beneficial predicting power of combining PTH and BSAP markers has not been completely verified yet.

### 4.3. TRAP5b

Tartrate-resistant acid phosphatase 5b (TRAP5b) is an enzyme secreted by osteoclasts to catabolize bone matrix. Therefore, elevated serum levels indicate enhanced osteoclastic activity [140], and studies in hemodialysis and late-stage CKD patients have demonstrated that TRAP5b correlates with bone turnover and resorption [141,142,143].

In non-dialysis patients, an early study identified significant negative correlations with renal function and a positive correlation with serum bone markers and the development of secondary hyperparathyroidism [144]. In a subsequent investigation, TRAP5b was an independent predictor of total hip BMD [136]. Furthermore, increased levels of TRAP5b have been associated with higher odds of fracture, and TRAP5b monitoring can discriminate fracture status in non-dialysis CKD patients independent of BMD [145].

An additional study reported a significant correlation between elevated TRAP5b concentrations, reduced BSAP levels and higher arterial stiffness, suggesting that the disparity between bone resorption and formation in CKD, inferred by the TRAP5b:BSAP ratio, is prognostic of vascular stiffness in non-dialysis patients [109]. TRAP5b may be a useful marker for serum bone resorption and cardiovascular risk, as it is not affected by renal dysfunction [144]. However, further studies are required to validate this marker in non-dialysis CKD patients.

### 4.4. P1NP

Procollagen type 1 N-terminal propeptide (P1NP) is released during the extracellular cleavage of type 1 collagen and is a marker of bone formation [146]. Higher levels of P1NP, in association with osteocalcin and TRAP5b, have been linked with a higher risk of fracture, even after adjustment for femoral neck T-score, suggesting that quantification of bone turnover markers may strengthen the diagnostic accuracy of densitometry to detect non-dialysis patients with CKD with a higher risk of fracture [145]. It is important to consider that both TRAP5b and the intact form of P1NP, in addition to alkaline phosphatases, do not have renal clearance [23].

### 4.5. Sclerostin

Sclerostin is a protein primarily produced by osteocytes, which plays a crucial role in regulating bone metabolism [147]. By binding to specific receptors on osteoblasts, sclerostin prevents the activation of Wnt/β-catenin signaling. This inhibits the differentiation and activity of osteoblasts, leading to a decrease in bone formation [148]. Sclerostin regulation is not widely understood. However, animal studies have demonstrated that PTH suppresses sclerostin expression in osteocytes [149]. In the setting of CKD, sclerostin levels have been reported to increase as renal function declines [150]. Elevated sclerostin levels in stage 3–4 non-dialysis patients and PTH resistance could contribute to the early appearance of low-turnover bone disease [129,150,151]. High-turnover bone disease will likely develop as CKD progresses and serum PTH levels overcome peripheral resistance [129]. While there is sufficient evidence to show that sclerostin impacts bone formation, further research on its potential as a biomarker for assessing bone health is warranted [152,153].

In addition, studies have established significant links between sclerostin levels and aortic, carotid and coronary artery calcification [148,154,155] in non-dialysis CKD, with one study suggesting that sclerostin measurement appears to be more important than serum phosphate levels for detecting VC-associated risk factors in patients with CKD who are not undergoing dialysis [148]. The association with VC may be important, as outlined in a further study demonstrating that serum sclerostin levels correspond with an increased risk of both fatal and non-fatal cardiovascular events in a non-dialysis CKD population, even after multiple statistical adjustments [156]. This is supported by two recent studies in stage 3 and 4 CKD, which identified a link between high sclerostin levels and low rates of bone turnover with a corresponding increase in VC [90,150]. The independent association between VC and the rate of bone formation highlights evidence of a key interplay between bone and the cardiovascular system in patients with CKD [90].

### 4.6. Bone Histology Tools

Up to half of patients with moderate kidney failure have irregular bone histology, suggesting that alterations to the skeleton may begin years before symptoms become apparent [157]. Bone biopsy is still considered the benchmark in assessing CKD-MBD as it offers evidence of bone mineralization, turnover and information regarding microstructural integrity [158,159]. Nevertheless, biopsy is seldom performed due to the invasive nature of the procedure, the potential risk of complications and the lack of experienced pathologists [15,16]. However, as biochemical data may not be sufficient to predict bone pathology in earlier stages of kidney disease, bone biopsy may be considered to define these bone changes and to allow appropriate therapeutic approaches in non-dialysis patients [160].

Assessment of bone mineral density (BMD) using dual-energy X-ray absorptiometry (DXA) is suggested by the current KDIGO guidelines for patients with stage 3–5 CKD showing evidence of CKD-MBD, as the lack of bone biopsy may not be sufficient justification for reserving antiresorptive therapy in patients with a high fracture risk [4]. Among non-dialysis patients, studies have shown that BMD measurement by DXA can discriminate fracture risk in stage 3–5 CKD and predict future fractures in patients with stage 1–3 CKD [161,162,163,164].

Although bone strength, inferred by its density, can be established by DXA, bone quality (as opposed to quantity) relating to factors such as remodeling, microdamage, and microarchitecture cannot be determined using this method [165]. Furthermore, a recent study suggested that DXA may overestimate lumbar spine BMD, likely due to an increased abdominal aortic calcification volume in non-dialysis patients [166].

Peripheral quantitative computed tomography (pQCT) can measure both trabecular and cortical volumetric density [165], and is considered a new tool for monitoring bone health in CKD. Several studies have demonstrated the utility of this technique to detect bone loss and accurately assess bone quality status [161,162,166], with a strong correlation to bone biopsy results [167], in non-dialysis patients. High-resolution pQCT (HR-pQCT) has a greater resolution than pQCT, which permits the assessment of trabecular number, thickness, separation and the direct quantification of cortical density, thickness, geometry and porosity at the distal radius and tibia, providing a greater indication of bone quality [165]. In non-dialysis patients, lower bone quality, assessed by HR-pQCT, is associated with an increased risk of fractures [161], and a progressive reduction in bone structural integrity has been observed from CKD stages 3–5 using this technique [168,169].

A further non-invasive measurement of bone structure can be achieved by determining the trabecular bone score (TBS), which evaluates texture variations and bone quality from images acquired from lumbar spine DXA [170]. Recent studies have demonstrated that lower TBS scores are associated with declining renal function and increased fracture risk and that the technique can accurately assess bone status in non-dialysis adult CKD patients [166,170]. Although the focus of this review is on markers and diagnostic tools in non-dialysis adult patients with CKD, bone histology tools are also used in pediatric studies. For example, a recent study used TBS scores to show that children and young adults with CKD stages 4 to 5 or on dialysis may develop VC even as their BMD increases [171]. Finally, another minimally invasive technique, impact microindentation, measures the resistance of bone tissue to an applied mechanical challenge via a probe inserted through the skin to the bone surface [172]. In a recent study, bone material strength index values were significantly lower in CKD patients receiving dialysis than in 94 healthy non-CKD controls [173]. However, no studies to date have validated the technique for assessment of bone quality in non-dialysis patients.

## 5. Conclusions

Despite advances in the field, optimal management of CKD-MBD and SHPT in non-dialysis adult CKD patients remains challenging. While there are established markers for prognosis and treatment response in late-stage kidney disease patients receiving dialysis, far fewer markers have been identified for CKD-MBD patients not receiving dialysis. Recent evidence highlights several promising tools and markers for the prediction of disease progression and risk of adverse clinical outcomes in non-dialysis CKD patients; however, many of them are not currently available in everyday clinical practice and further research is needed to define parameters that inform optimal clinical management or likely treatment responses.

Cardiovascular and bone disease are common complications in CKD patients, highlighting the complex relationship between bone turnover and vascular calcification in CKD patients. In addition to biochemical markers, bone and vascular imaging should be considered important tools to guide treatment decisions for non-dialysis CKD patients and the focus of future research.

## Figures and Tables

**Table 1:** Summary of marker roles relating to CKD-MBD.

**General Mineral Dysregulation Markers**	
Phosphate	Essential for bone growth and mineralization; multiple key roles in cellular maintenance, metabolism and signaling
Parathyroid hormone	Regulates serum calcium and phosphate concentration through effects on the gastrointestinal tract, kidneys and bone
Calcium	Essential for bone formation; versatile signaling molecule with multiple physiological functions
25-hydroxyvitamin D (25(OH)D)	The main circulating form of vitamin D; serves as a precursor to the active form of vitamin D—1,25-dihydroxyvitamin D (1,25(OH)_2_D)
1,25-dihydroxyvitamin D (1,25(OH)_2_D)	Increases absorption of calcium from the gastrointestinal tract and promotes its reabsorption in the kidneys; helps regulate phosphate levels; suppresses the release of PTH from the parathyroid glands
Fibroblast growth factor-23 (FGF-23)	Suppresses phosphate reabsorption and calcitriol synthesis in the kidney
Klotho	Obligate co-receptor for FGF-23; role in the regulation of survival and vascular calcification
**Cardiovascular Dysfunction Markers**	
Matrix Gla protein	Helps regulate vascular calcification by scavenging circulating calcium phosphate and interacting with fetuin-A to form primary calciprotein particles
Fetuin-A	Helps regulate vascular calcification by binding to calcium phosphate and interacting with matrix Gla protein to form primary calciprotein particles
Primary calciprotein particles (CPPs)	Develop into secondary CPPs, which contribute to vascular calcification
Secondary calciprotein particles (CPPs)	Are involved in the initiation and progression of vascular calcification
Osteocalcin	Promotes bone mineralization and plays a multifaceted role in cardiovascular health by influencing insulin sensitivity, vascular function, cardiac function and the regulation of calcification processes
**Markers of Bone Turnover**	
Bone-specific alkaline phosphatase	Contributes to the process of bone turnover by regulating bone matrix mineralization
Tartrate-resistant acid phosphatase 5b (TRAP5b)	Released by osteoclasts to break down bone matrix
Procollagen type 1 N-terminal propeptide (P1NP)	A marker of bone formation released following extracellular cleavage of type 1 collagen
Sclerostin	Inhibits bone formation by osteoblasts and influences vascular calcification

**Table 2:** Summary of tools available for use in the diagnosis and management of CKD-MBD.

**Tools that Assess Cardiovascular Health**	
Coronary artery calcification (CAC) score	Uses computed tomography to measure the amount of calcified plaque in the coronary arteries
Kauppila score	Uses lateral lumbar radiographs to quantify abdominal aortic calcification
Adragao score	Uses X-rays of the pelvis and hands to assess vascular calcifications of iliac, femoral, radial and digital arteries
Pulse wave velocity	Is a measure of arterial stiffness. It is calculated by measuring the time it takes for the arterial pulse to travel between two points (e.g., carotid and femoral arteries) divided by the distance between those points
**Non-Invasive Tools that Assess Bone**	
Dual-energy X-ray absorptiometry (DXA)	Uses spectral imaging to measure bone mineral density
Peripheral quantitative computed tomography (pQCT)	Is a 3D imaging technique used to quantify bone mineral density in peripheral skeletal sites, e.g., spine, proximal femur, forearm and tibia
Trabecular bone score (TBS)	Uses images acquired from lumbar spine DXA to evaluate bone texture variations and bone quality

## Data Availability

No new data were generated or analyzed for this review article. Data sharing is not applicable.
